# The study of the relation of DNA repair pathway genes SNPs and the sensitivity to radiotherapy and chemotherapy of NSCLC

**DOI:** 10.1038/srep26526

**Published:** 2016-06-01

**Authors:** Chunbo Wang, Huan Nie, Yiqun Li, Guiyou Liu, Xu Wang, Shijie Xing, Liping Zhang, Xin Chen, Yue Chen, Yu Li

**Affiliations:** 1 School of Life Science and Technology, Harbin Institute of Technology, Heilongjiang, China; 2 Department of Radiotherapy, Affiliated Tumour Hospital of Harbin Medical University; 3 Genome Analysis Laboratory, Tianjin Institute of Industrial Biotechnology, Chinese Academy of Sciences, China; 4 Department of Radiotherapy, Beijing Miyun County Hospital.

## Abstract

To analyze the relation between SNPs in DNA repair pathway-related genes and sensitivity of tumor radio-chemotherapy, 26 SNPs in 20 DNA repair genes were genotyped on 176 patients of NSCLC undertaking radio-chemotherapy treatment. In squamous cell carcinoma (SCC), as the rs2228000, rs2228001 (XPC), rs2273953 (TP73), rs2279744 (MDM2), rs2299939 (PTEN) and rs8178085, rs12334811 (DNA-PKcs) affected the sensitivity to chemotherapy, so did the rs8178085, rs12334811 to radiotherapy. Moreover rs344781, rs2273953 and rs12334811 were related with the survival time of SCC. In general, the “good” genotype GG (rs12334811) showed greater efficacy of radio-chemotherapy and MSF (24 months) on SCC. In adenocarcinoma, as the rs2699887 (PIK3), rs12334811 (DNA-PKcs) influenced the sensitivity to chemotherapy, so did the rs2299939, rs2735343 (PTEN) to radiotherapy. And rs402710, rs80270, rs2279744 and rs2909430 impacted the survival time of the adenocarcinoma patients. Both GG (rs2279744) and AG (rs2909430) showed a shorter survival time (MFS = 6). Additionally, some SNPs such as rs2228000, rs2228001 and rs344781 were found to regulate the expression of DNA repair pathway genes through eQTLs dataset analysis. These results indicate that SNPs in DNA repair pathway genes might regulate the expression and affect the DNA damage repair, and thereby impact the efficacy of radio-chemotherapy and the survival time of NSCLC.

The International Agency for Research on Cancer announced in 2012 that lung cancers were still the most common cancers in the world (1.8 million cases, accounting for 13% of all new cases) and the leading cause of cancer deaths (1.6 million deaths, accounting for 19.4% of all cancer deaths)^1^. In China, the incidence and mortality rate of lung cancers are also the highest among all cancers. In 2009, statistics showed that the number of new cases of lung cancers reached 600,000, approximately 490,000 patients died each year, and these numbers showed a trend to increase every year^2^. Non-small cell lung cancer (NSCLC) accounts for approximately 80% of all lung cancers. At present, surgery is still the most effective treatment for lung cancers. However, due to the development of the diseases and tumor sizes, the number of suitable cases for surgery is low. Approximately 70–80% of patients need to be treated with radio-chemotherapy either alone or after surgery. The 5-year survival rates of stage III and IV patients vary from 5% to 15%^3^. Even among patients with same pathology type and same clinical stage, there are huge differences in treatment efficacy exist among the different individuals.

The main mechanism of radio-chemotherapy is to induce DNA damages in the tumor cells, leading to the irreversible death of these cells. As an important mechanism to maintain genome stability and to repair damaged DNA, DNA repair machinery plays an important role in tumor genesis, development, metastasis and prognosis. Studies have indicated that the enhancement of DNA damage repair ability can effectively reduce the occurrence of tumor^4^. However, while this enhancement provides genome stability, importantly, it can also result in tolerance of the tumor cells toward radio-chemotherapy and to a reduced efficacy of clinical treatments^5^.

The responses of tumor cells to DNA damages are involved with very complicated molecular regulation networks, and tumor cells also possess self-repair capacities. The multiple pathways and repair methods of this mechanism can affect the damage repair and survival of tumor cells. Radiotherapy induces tumor cell apoptosis mainly through the production of free radicals, which induce double-stranded DNA breaks, crossovers and oxidizing damages to nucleotide bases. These changes will activate the DNA damage response (DDR), whose main repair mechanisms are homologous recombination repair (HR) and nonhomologous end joining (NHEJ)^6^. Platinum-containing anticancer drugs can result in DNA intrastrand and interstrand crossovers in target cells to inhibit DNA synthesis and replication, and thereby inhibit the growth of tumor cells. The main repair mechanism of these damages is nucleotide excision repair (NER)^7^. In additional, other DNA repair pathway-related genes also contribute to these responses^8^. Therefore, it is important to understand the roles of DNA repair pathway-related genes in the radio-chemotherapy of tumors.

Single nucleotide polymorphisms (SNPs) represent the third generation of molecular markers of genetic variation. SNPs are used mainly to study disease susceptibility and differences insensitivity to drugs and treatment methods among different individuals. Genome-wide association study (GWAS) is a new tool to provide a mechanism to assess variation of SNP. Now, it has been widely used to study the biomarkers for the survival, advanced, and susceptibility of the NSCLC by using high-throughput genotyping technology and selecting tagging SNPs across the whole genome. Until now, 13 NSCLC GWASs have been conducted to identify common susceptibility SNPs for NSCLC^9^,^10^,^11^, response to irinotecan in NSCLC^12^, NSCLC survival^13^,^14^, NSCLC recurrence rate^15^, response to platinum-based chemotherapy in NSCLC^16^,^17^, response to irinotecan and platinum-based chemotherapy in NSCLC^18^, and lung cancer (DNA repair capacity)^19^. These 13 GWASs have investigated NSCLC pathogenesis, and yielded important new insights into the genetic mechanisms of NSCLC.

Genetic variation in DNA damage repair genes is a universal phenomenon in living organisms, which can change the repair capacities of individuals to DNA damages and then affect the survival status of tumor patients^20^. Many studies have revealed the involvement of SNPs in DNA damage repair-related genes in lung cancer susceptibility^21^, and some have reported the relation between SNPs and sensitivity to radio-chemotherapy^22^ as well as the GWAS^19^. However, the results are inconsistent, and either about a single gene or about a single pathway or on the GWAS level^19^. However, there are little comprehensive study on the SNP of DNA repair genes and the sensitivity to radio-chemotherapy. Therefore, based on the assumption that SNPs of genes in multiple related pathways affect DNA repair, this study sought to analyze the correlation between SNPs and the outcomes of radio-chemotherapy, discuss the differential treatment efficacy of non-small cell lung cancers based on different genotypes of SNPs in DNA damage repair pathway genes and provide reliable support for clinical treatment regimens. Meanwhile, in order to validate the effect of these variant on gene expression, we performed an expression quantitative trait loci (eQTLs) analysis using three large-scale expression datasets.

## Results

### Screening of SNPs in DNA damage repair-related genes

To extensively explore the relation between DNA damage repair and the efficacy of radio-chemotherapy of lung cancers, this study analyzed the SNPs in DNA damage repair-related genes. The SNP database (dbSNP) of National Center for Biotechnology Information (NCBI, http://www.ncbi.nlm.nih.gov/snp/) and related literature were selected to identify functional SNPs. Based on the main mechanisms of DNA repair and the important repair-related pathways, we finally selected 26 SNPs in 20 related genes for following analysis. Detailed information about the 26 SNPs is listed in Table 1.

### Patient clinical features and the treatment efficacy

A total of 176 patients were enrolled, with a male to female ratio of 2.4:1. The median patient age was 58 (23–88 years old). There were 106 cases of squamous cell carcinoma and 70 cases of adenocarcinoma. The Eastern Cooperative Oncology Group (ECOG) scores were 0–2. The overall 1-year and 3-year survival rates were 80.5% and 27.4%, respectively, for the whole group. The median overall survival time (OS) was 22 months. The progression-free survival time (PFS) rates of 1 year and 3 years were 53.0% and 20.8% respectively, and the median PFS was 12 months.

From the clinical features and treatment effects of squamous cell carcinoma (Table S1), no obvious correlation was revealed between the patients’ sex, age, sequence of treatment arrangement and preferred chemotherapy regimen with the PFS and OS in patients. However, different clinical stages and different sensitivity to radio-chemotherapy led to significant differences in both PFS and OS. Clinical stages are correlated with the development of disease, and sensitivity to radio-chemotherapy represents the influence of tumor intrinsic factors that affect treatment efficacy. Therefore, at the same clinical stage, sensitivity to radio-chemotherapy is the definitive factor for determining PFS and OS.

Similar to the situations of squamous cell carcinoma, the main factors affecting PFS and OS in adenocarcinoma patients were also clinical stages and sensitivity to chemotherapy. As a means of localized treatment, the sensitivity to radiotherapy did not reveal a significant difference. This phenomenon is related to the biological characteristics of adenocarcinomas, which include low sensitivity, being prone to distant metastasis, etc. (Table S2).

### Correlation analysis between the sensitivity to radio-chemotherapy and SNPs in squamous cell carcinoma

The sensitivity to radio-chemotherapy of an individual patient is the decisive factor of survival time after radio-chemotherapy. SNPs are an important index for use in studying the difference in sensitivities toward drugs and treatments among different individuals. Table 2 showed the correlation analysis results about the radio-chemotherapy treatment efficacy of 90 squamous cell carcinoma patients and 26 SNPs in DNA damage repair-related genes.

The results showed that the SNPs in XPC (rs2228000 and rs2228001), TP73 (rs2273953), MDM2 (rs2279744), PTEN (rs2299939) and DNA-PKcs (rs8178085 and rs12334811) genes significantly affected the sensitivity of lung squamous cell carcinoma to chemotherapy. With the chemotherapy treatment of squamous cell carcinoma, among patients of the same age and clinical stage, patients with non-CC homozygous genotypes of the SNP rs2228000 (XPC) showed higher chemotherapy efficacy (OR = 3.94) compared with the CC homozygous genotype patients. Patients with the AC heterozygous genotype of the SNP rs2228001 (XPC) showed better chemotherapy efficacy than patients with a CC homozygous genotype (OR = 6.17). Patients with a CC homozygous genotype of the SNP rs2273953 (TP73) demonstrated curve effect than patients with a CC homozygous genotype (OR = 17.67). Patients with non-TT homozygous genotypes of the SNP rs2279744 (MDM2) showed higher chemotherapy efficacy than patients with a TT homozygous genotype (OR = 4.54). Patients with a CC homozygous genotype of the SNP rs2299939 (PTEN) is better than patients with a non-CC homozygous genotype (OR = 4.29). Patients with a GG homozygous genotype of the SNP rs12334811 (DNA-PKcs) (in the non-AG heterozygous genotypes, only one case of an AA homozygote was detected) revealed better impact than patients with an AG heterozygous genotype (OR = 4.22). Additionally, patients with a CA heterozygous genotype of SNP rs8178085 (DNA-PKcs) were completely insensitive to chemotherapy.

With the radiotherapy treatment of squamous cell carcinoma, we found that the rs12334811 and rs8178085 were sensitive to radiotherapy. Patients with a GG homozygous genotype of rs12334811 (DNA-PKcs) showed higher radiotherapy efficacy than patients with an AG heterozygous genotype (OR = 4.97). Patients with an AA homozygous genotype of rs8178085 (DNA-PKcs) is better effect than patients with non-CA heterozygous genotypes (OR = 9.49).

We found that many SNPs in DNA damage-repair genes were correlated with squamous cell carcinoma sensitivity to radio-chemotherapy. Among them, the GG homozygous genotype of rs12334811 and the AA homozygous genotype of rs8178085 of the DNA-PKcs gene belong to “good” genotypes, which demonstrate significant sensitivity to radio-chemotherapy.

### Correlation analysis between sensitivity to radio-chemotherapy and SNPs in adenocarcinoma

We also studied the effects of SNPs in adenocarcinoma (Table 3). Through the correlation analysis of radio-chemotherapy treatment efficacy in 63 adenocarcinoma patients and SNP sites on DNA repair-related genes, we found that in chemotherapy treatment of adenocarcinoma, SNPs in PIK3 (rs2699887) and DNA-PKcs (rs12334811) significantly affected the sensitivity to chemotherapy. Patients with an AG heterozygous genotype of rs2699887 (PIK3) showed higher chemotherapy efficacy than GG homozygous genotype (OR = 14.98). Patients with an AG heterozygous genotype of rs12334811 (DNA-PKcs) is better than GG homozygous genotype (OR = 4.63).

In radiotherapy treatment of adenocarcinoma, we found that rs2299939 and rs2735343 in PTEN gene were sensitive to radiotherapy of adenocarcinoma. Patients with a non-CC homozygous genotype of rs2299939 (PTEN) showed higher radiotherapy efficacy than CC homozygote genotype (OR = 15.64). Patients with a non-CC homozygous genotype of rs2735343 (PTEN) is better than CC homozygous genotype (OR = 6.13). Detailed results are shown in Table 4.

The above results suggest that the PIK3 gene showed sensitivity to chemotherapy and the PTEN gene showed sensitivity to radiotherapy in adenocarcinoma, indicating that the effects observed in adenocarcinoma treatment result from the cooperative effects of multiple genes of a certain pathway, rather than a single gene.

### Correlation analysis between SNPs and the survival time of patients with different pathological types of lung cancer

On the basis of the study about the correlation of SNPs with the sensitivity to radio-chemotherapy, we proceeded with correlation analysis between SNPs and the survival time of patients.

Three SNPs (rs344781, rs2273953 and rs12334811) have a significant effect on the survival of patients with squamous cell carcinoma (Table 4). Among patients of the same age and clinical stage, patients with a CC homozygous genotype of rs2273953 had longer OS than TT homozygous genotype (*P* < 0.05) and longer PFS than CT heterozygous genotype (*P* < 0.05). Patients with a GG homozygous genotype of rs12334811 had longer OS than AG heterozygous genotype (*P* < 0.01). Patients with a TT homozygous genotype of rs344781 had longer PFS than CC homozygous genotype (*P* < 0.05).

Meanwhile, we found that patients with a GG homozygous genotype of rs2273953 (DNA-PKcs) had significant sensitivity to radio-chemotherapy of squamous cell carcinoma, and patients with a CC homozygous genotype of rs12334811 (TP73) showed significant sensitivity to chemotherapy of squamous cell carcinoma. These genotypes also showed a longer survival time. The results indicate that the SNPs in these two genes may have important implications for the treatment and prognosis of squamous cell carcinoma.

The correlation analysis of SNPs and the survival time of all adenocarcinoma patients revealed that among patients of the same age and clinical stage, patients with a CC homozygous genotype of rs402710 (CLPTM1L) had longer PFS compared with CT heterozygous genotype (*P* < 0.05).Patients with an AA homozygous genotype of rs80270 (CDKN1A) prolong PFS compared with patients with a CC homozygous genotype (*P* < 0.05). Patients with a GT heterozygous genotype of rs2279744 (MDM2) had longer PFS and OS (*P* < 0.05). Patients with an AA homozygous genotype of rs2909430 (TP53) had better OS compared with patients with an AG heterozygous genotype (*P* = 0.016), as shown in Table 4.

Among the SNPs related to the survival time of adenocarcinoma patients, MDM2 and TP53 significantly affected the survival time of the adenocarcinoma patients. Together with earlier results showing that PIK3 had sensitivity to chemotherapy in adenocarcinoma and PTEN had sensitivity to radiotherapy in adenocarcinoma, these findings suggest that the polymorphisms in PTEN, PI3K, Mdm2 and p53 genes, which belong to a functionally related network, may function cooperatively in the radio-chemotherapy and survival time of adenocarcinoma.

### Correlation analysis of SNPs jointly participated in the regulation of survival time of different pathological types of lung cancers

The effects of SNPs on the radio-chemotherapy and survival time of lung cancers might be joint actions of multiple variables. Based on results from single SNP, we further performed joint multivariate analysis on a number of genotypes with survival advantages.

**Figure 1:**
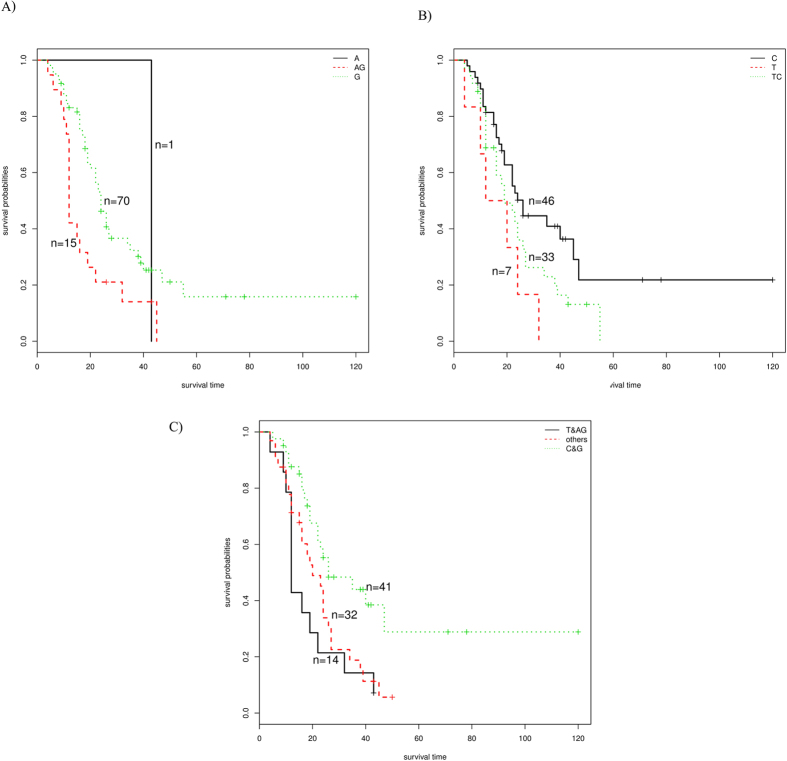
Stepwise regression analysis results of SNP rs2273953 (TP73) and rs12334811 (DNPK1) in squamous cell carcinoma patients (**A**) rs12334811(DNPK1) (**B**) rs2273953(TP73) (**C**) Conjoint analysis of rs12334811(DNPK1)and rs2273953(TP73).

In the analysis of squamous cell carcinoma, we chose three SNPs with longer survival time, rs344781 (PLAUR, TT homozygote), rs2273953 (TP73, CC homozygote) and rs12334811 (DNPK1, GG homozygote), to do stepwise regression analysis, and the results are shown in Fig. 1. Patients with both rs2273953 (TP73) CC and rs12334811 (DNPK1) GG SNPs, compared with a single SNP, could be better distinguished by survival time (*P* = 0.018), although the median survival time did not change (MSF = 24).

**Figure 2:**
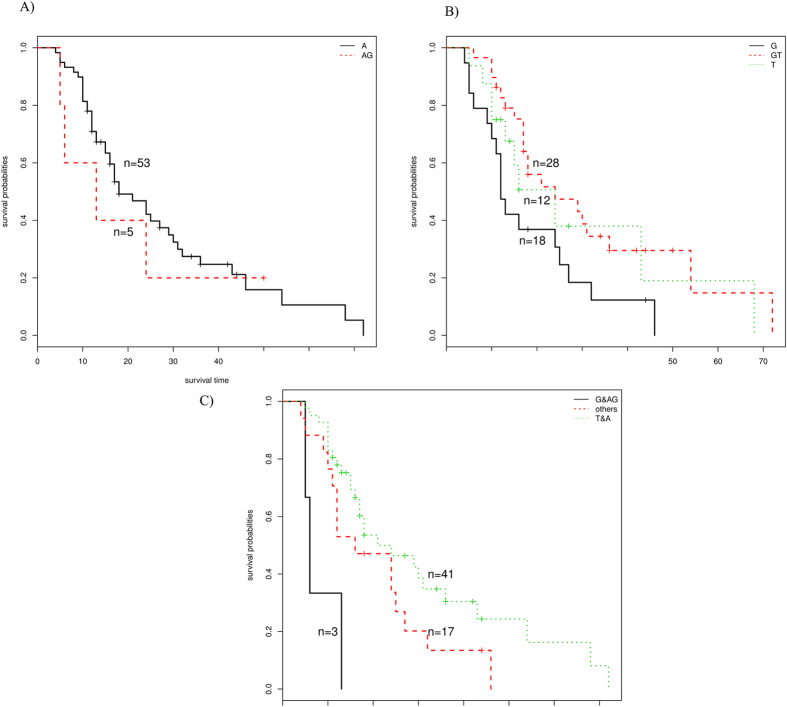
Stepwise regression analysis results of SNP rs2279744 (MDM2) and rs2909430 (TP53) in adenocarcinoma patients. (**A**) rs2909430(TP53) (**B**) rs2279744(MDM2) (**C**) Conjoint analysis of rs2909430(TP53)and rs2279744(MDM2).

In the analysis of adenocarcinoma, we performed stepwise regression analysis on four SNPs showing significant correlation with OS: rs402710 (CLPTM1L), rs1801270 (CDKN1A), rs2279744 (MDM2) and rs2909430 (TP53). We found that the combination of the two “worst” genotypes, rs2279744 (MDM2, GG homozygote) and rs2909430 (TP53, AG heterozygote), showed significant differences in their effects on survival time (Fig. 2). Compared with single SNP, the median survival time of patients with these two genotypes was greatly decreased (MSF = 6; *P* = 0.018).

The above results indicate that in non-small cell lung cancers, rs2273953 (TP73), rs12334811(DNPK1), rs2279744 (MDM2) and rs2909430 (TP53) can be used as biological markers for clinical treatment and prognosis.

### eQTLs analysis

It is widely acknowledged that SNPs are not only one of the genetic markers, but it can also regulate the expression of related genes. Using the eQTLs dataset from peripheral blood samples, we found that 10 of the 26 selected SNPs could significantly regulate the expression of 15 genes as described in Table 5. Among the 10 SNPs, 8 SNPs including rs2228001, rs2228000 and rs344781 are associated with the expression of DNA damage repair-related genes. We also discovered that the rs2228001 (XPC) and rs2228000 (XPC) were relevant to the sensitivity of chemotherapy, and rs344781 (PLAUR) was related to the survival of patients of lung squamous cell carcinoma through the above research (Tables 2 and 4). The results showed the reason for the sensitivity of radio-chemotherapy is probably that the rs2228000, rs2228001 and rs344781 induced the variation of gene expression of XPC and PLAUR. Furthermore, rs1130214, rs11615, rs159153 and rs2132572 can regulate the expression of AKT, ERCC1, OGG1 although they have no relation with chemo-radiotherapy.

Using the eQTLs dataset from lymphoblastoid cell lines, we identified three significant associations between three SNPs (rs2228001, rs25406 and rs8073069) and the expression of XPC, C20orf30 and BIRC5 as described in Table 6.

Using the GTEx samples in human lung tissue, we identified two SNPs including rs25487 and rs25406, which could significantly regulate ZNF575 (*P* = 1.50E-07 and effect size = 0.30) and TMEM230 (*P* = 9.40E-11 and effect size = 0.18), respectively.

In summary, our results revealed that the SNPs in DNA repair pathway genes might regulate the gene expression and affect the DNA damage repair.

## Discussion

At present, combined radio-chemotherapy is still the standard treatment regimen for local advanced non-small cell lung cancers. Despite advances in radiotherapy techniques and the development and application of a new generation of chemotherapy drugs, the efficacy of radio-chemotherapy is still hovering in the range of 5–15%^8^. The reason for this low efficacy is mainly due to individual differences; that is, patients with similar clinical features demonstrate different treatment outcomes. Therefore, we performed regression analysis to investigate the potential association of SNPs in DNA damage repair-related genes with patient survival time and sensitivity to radio-chemotherapy separately. We found that a large number of SNPs in DNA damage repair-related genes were associated with the sensitivity to radio-chemotherapy, some SNPs also affected the survival time of patients, and the joint actions of multiple sites could further enhance the effect on survival time. In addition, some SNPs could regulate the expression of DNA repair pathway genes through the eQTLs dataset analysis.

In squamous cell carcinoma, we found that the GG genotype of rs12334811 in the DNA-PKcs gene had a significant effect on sensitivity to radio-chemotherapy and longer OS. DNA-PKcs is an important gene of NHEJ (nonhomologous end joining), which is an important pathway for double stranded break repair, functioning in all cell cycle phases and accounting for approximately 90% of DNA repair^23^. DNA double strand breaks are lethal damages in cells and can directly affect the efficacy of radio-chemotherapy. Hu *et al*. found that rs12334811 was associated with sensitivity to treatments of lung cancer^24^. We also found that the GG genotype of rs12334811 in the DNA-PKcs gene had significant importance on radio-chemotherapy treatments and survival time in squamous cell carcinoma, showing a median survival time of 24 months. Mutations of this gene may affect DNA repair ability, thereby affecting the efficacy of radio-chemotherapy, and eventually affecting the survival time of patients. Xing *et al*. examined the ratios of tumor to surrounding normal tissues of ATM and DNA-PKcs separately^25^. Many researchers have reported the role of DNA-PKcs in treatments of other cancers, such as in prostate cancers^26^. Multivariate analysis showed that the Gleason score, PSA level and DNA-PKcs status could be regarded as the predictors of recurrence. In nasopharyngeal carcinoma, a low expression level of DNA-PKcs in tumor tissues was related to a high metastasis rate and therefore is a marker of poor prognosis^27^. Through histology studies, Hu *et al*. further indicated that patients with a low expression level of DNA-PKcs could benefit more from radiotherapy^28^. Some studies have proven that NHEJ is the main pathway of DNA double strand break (DSB) repair^29^. DNA-PKcs is not only one important regulatory factors of NHEJ but also maintains the stability of the genome, monitors the process of mitosis and regulates HR. Many small molecular reagents target DNA-PK by inhibiting NHEJ repair, decrease the DNA damage-repair ability after radio-chemotherapy, increase sensitivity to radio-chemotherapy and achieve the purpose of the tumor treatments. In this study, the SNP site rs12334811of the DNA-PKcs gene was closely related with squamous cell carcinoma sensitivity to radio-chemotherapy. This genotype may increase the sensitivity of patients to radio-chemotherapy of squamous cell cancers, enhance treatment efficacy and prolong the survival time of the patient.

We also found that the CC homozygous genotype of the rs2273953 in the TP73 gene had significant importance in chemotherapy and survival time of squamous cell carcinoma. As a member of the P53 family, P73 has been found to be involved in many biological processes such as cell proliferation, apoptosis, development, differentiation, senescence, and aging^30^. Zaika *et al*. found that p73 played a very important role in the repair of DNA damages^31^. Zhou *et al*. revealed that the polymorphisms G4C14-to-A4T14 of p73 (rs2273953, rs1801173) had a positive correlation with triple-negative breast cancer (TNBC) and then found that it could be used in the treatment of TNBC^32^.We found that the CC homozygous genotype of the rs2273953 in the TP73 gene was sensitivity toward chemotherapy in squamous cell carcinoma and had a median survival time of 26 months compared with other genotypes (*P* < 0.05). This indicates that rs2273953 may affect DNA damage-repair ability to some extent, which may induce sensitivity to chemotherapy and then prolong the survival time of the patients.

In adenocarcinoma, we found that the rs2699887 in PI3K had a great effect on the chemotherapy of adenocarcinoma. Two SNPs rs2299939 and rs2735343 in PTEN gene had significant importance in the radiotherapy of adenocarcinoma (*P* < 0.05). The rs1801270 in CDKN1A gene, rs2279744 in MDM2 gene and rs2909430 in TP53 gene were related with the survival time of adenocarcinoma patients (*P* < 0.05). The effects of these polymorphisms suggested a close relationship between PI3K, PTEN, Mdm2 and p53 and the efficacy of radio-chemotherapy treatment of adenocarcinoma and the survival time of patients. PTEN, PI3K, Mdm2 and p53 belong to a network of oncogenes and antioncogenes and are related to many important physiological functions of the cell^33^. Liu *et al*. observed SNPs in P53 (rs1042522) and MDM2 (rs2279744) in 199 patients with stage III – IV non-small cell lung cancer who were undergoing cisplatin-based chemotherapy^34^. The results showed patients with Pro/Pro genotype of P53 (rs1042522) combined with the GG genotype of MDM2 (rs2279744) had a survival time only half that of the patients with the wild-type genotype. By analyzing the 16 SNPs in the PI3K-PTEN-AKT-mTOR pathway, Li *et al*. found that SNPs in this pathway were closely correlated with brain metastasis of NSCLC^35^. Ming *et al*. reported that the down regulation of PTEN could enhance cell growth inhibition and activate Chk1DNA damage pathway in an AKT-dependent way^36^. The absence of PTEN inhibits the expression of XPC, a GG-NER pathway key factor. Using NSCLC cell lines, Fan *et al*. proved that PTEN pathway affected the efficacy of vinorelbine^37^. The clinical studies conducted by Pu *et al*. found that the SNP site rs2699887 of PIK3 could increase the toxicity of treatments, whereas the SNP sites of PTEN could alleviate the toxicity of chemotherapy^38^. In our study, PTEN (rs2299939, rs2735343), PI3K (s2699887), MDM2 (rs2279744) and P53 (rs1042522) significantly affected the chemotherapy sensitivity and survival time of lung adenocarcinoma patients, suggesting that the related genes of this pathway and the combined effects of these genes changed the tumor sensitivity to radio-chemotherapy, and then changed the survival time after treatment of NSCLC. These results suggest that the SNPs in related genes of this pathway can be used as markers for follow-up individualized treatments.

In addition, we performed combined analyses of multiple SNPs. We found that, in patients with squamous cell carcinoma, the survival time of patients with the GG genotype of rs12334811 and CC genotype of rs2273953 was significantly different compared to other groups (*P* = 0.018). In patients with adenocarcinoma, the median survival time of patients with the GG genotype of rs2279744 and the AG genotype of rs2909430 was only 6 months (*P* = 0.018), indicating that the combination of these two genotypes predicts poor prognosis. Although there are only a few of cases (rs2279744 homozygote and rs2909430 AG heterozygote), it shows great value in prognostic effects. And more researches are required to confirm that. Therefore, SNPs in these genes are likely to have a role in regulating the function of these genes. And different genes in the same pathway and genes of different pathways also have certain synergistic effects. As a result, the combination of different genotypes results in differences in radio-chemotherapy treatment efficacy and affects the survival time after treatment of NSCLC. The combined detection of the related sites may become an effective method for judging the efficacy of radio-chemotherapy in non-small cell lung cancer.

In this study, we also noticed that the same genotype of the same SNP had different effects on treatment efficacy in squamous cell carcinoma or adenocarcinoma, and there were different synergistic effects between different SNP genotypes. In addition to the complex network formed by the DNA repair pathways, a network intertwined by SNP genotypes, pathological types and different individuals is further formed. The results of this study show that the complexity of biological systems determines that the efficacy of radio-chemotherapy is not decided by one or more factors, but rather by the complex interaction of multiple factors.

GWASs were used to analyze the relation between SNP and the NSCLC. There are 13 NSCLC GWAS, which have investigated NSCLC pathogenesis, and yielded important new insights into the genetic mechanisms of NSCLC. Until now, 47 significant SNPs with *P* < = 1.00E-05 have been identified as described in supplementary Tables. However, these 47 SNPs are not in our research. Maybe the reason is that we focused on the relation between the SNPs and efficacy of radio-chemotherapy while those GWAS researches were focused on the survival, advance and chemotherapy, or the different population.

Additionally we found that the rs2228001 (XPC) and rs2228000 (XPC) were related with the sensitivity of chemotherapy and rs344781 (PLAUR) was related with the survival of patients of lung squamous cell carcinoma by eQTLs analysis. It has been reported that these SNPs could regulate gene expression^39^. These results revealed that the variation of SNP really could bring about the variety of gene expression and gene function of DNA damage repair.

We all know that more samples could provide more data and statistical power to avoid the effect of individual differences. In this study, only 176 NSCLC serums received radio-chemotherapy treatment were enrolled. Although the number in our research is not very large, the restrict treatment process and detailed follow up could be ensured. In future, more serum samples shall be done to confirm the relation between the SNPs in DNA repair genes and sensitivity to radio-chemotherapy.

## Conclusions

In the present study, SNPs in DNA damage repair-related genes showed a significant impact on the efficacy of radio-chemotherapy treatment of non-small cell lung cancer. The GG genotype of rs12334811 in the DNA-PKcs gene had a significant effect on radio-chemotherapy and survival time of lung squamous cell carcinoma, and the CC genotype of rs2273953 in the TP73 gene had a significant effect on chemotherapy and the survival time in patients with lung squamous cell carcinoma. Polymorphisms in the PTEN (rs2299939, rs2735343), PI3K (rs2699887), MDM2 (rs2279744), and P53 (rs1042522) genes influence the efficacy of radio-chemotherapy and survival time of lung adenocarcinoma. The combination of rs2279744 (MDM2) and rs2909430 (TP53) showed poor survival time. In addition, the.

These SNPs can be used as biomarkers for the treatment and prognosis of non-small cell lung cancer. The results of this study will provide a theoretical basis and data support for further research on the relations between SNPs and radio-chemotherapy as well as for the development of clinical treatment plans.

## Materials and Methods

### Patients

176 patients with non-small cell lung cancer were collected from Affiliated Tumor Hospital at the Harbin Medical University between March 2010 and March 2012. All patients were confirmed by pathology. All patients were followed up to August 2013, and the follow-up rate was 89.3%. The research was approved by the Ethical Committee of the Tumor Hospital of Harbin Medical University, and was in accordance with the ethical standards laid down in the 1964 Declaration of Helsinki. The informed consents signed by all the patients were gotten already. Before a patient was treated, a 5-ml sample of fasting vein blood was taken for DNA extraction. Genomic DNA was isolated from peripheral blood lymphocytes by the routine phenol–chloroform method and stored at −80 °C. Qualified patient DNA samples were used for SNP genotyping.

### Treatment regimens

A radiotherapy-based comprehensive treatment plan was employed. The radiotherapy plan is a three-dimensional conformal intensity-modulated treatment that requires irradiation of the involved area with conventional segmentation at 1.9Gy-2.0Gy/time, with the total dosage of 60Gy. Chemotherapy employed a Cisplatin-based two-drug regimen, which included Cisplatin with the third-generation chemotherapy drug vinorelbine (NP regimen), paclitaxel or docetaxel (TP regimen), gemcitabine (GP regimen) and pemetrexed (PP regimen), respectively, for a total of 4–6 cycles. All patients completed treatment (Tables S1 and S2).

### SNP detection method

Each DNA sample was diluted to working concentrations of 50ng/μl for genotyping. Assay design and SNP genotyping were performed by CapitalBio (Beijing, China) using the Sequenom MassARRAY platform (Sequenom, San Diego, CA). Primers were designed by Genotyping Tools and MassARRAY Assay Design software (version 3.0, Sequenom Inc., San Diego, California). SNPs were genotyped using the Sequenom MassARRAY iPLEX platform. Data was processed and analyzed by Sequenom MassArray TYPER 4.0 software.

### Clinical observation indices

Observations were recorded for progression-free survival time (PFS), overall survival time (OS) and radio-chemotherapy sensitivity (according to the efficacy evaluation standard of solid tumor RECIST, edition 1.1, using a CT scan). Sensitive is defined as at least a 30% reduction of the maximum diameter of the tumor after treatment, and otherwise is defined as nonsensitive). Other indices are listed in Tables S1 and S2

### Statistical analysis

First, we used a Cox regression model to perform a univariate survival analysis on lung adenocarcinoma and lung squamous cell carcinoma patients separately, using totally 8–13 clinical indices, including age, sex, clinical stage and others. In addition, in the survival analysis of the 26 SNPs, taking the age and the clinical stage of the patients as covariates, we analyzed separately the effect of the different genotypes of each SNP on the survival time of patients. Second, we used the logistic regression model to analyze the relation between SNPs and sensitivity to radio-chemotherapy, calculated the OR values of each SNP to describe the relation between the different genotypes and the efficacy of radio-chemotherapy, and also took the age and the clinical stage of the patients as covariates to eliminate the influence of mixing factors on results. Because all of the subjects were cancer patients, the sample numbers of the specific genotypes of some SNP sites were very small, and in those cases, the genotypes were categorized as other genotypes in the analysis. Finally, we did backward stepwise regression analysis on multiple SNP that significantly affect the OS of patients and combined the different genotypes of multiple genes to analyze the effects of multiple SNPs on the survival of patients. All of the above analyses were completed using R software version 3. At the same time, all *P* values were bilaterally tested.

### eQTLs analysis

Here, we selected three large-scale expression quantitative trait locus (eQTL) datasets from the non-transformed peripheral blood samples (5,311 individuals with replication in 2,775 individuals)^39^, the lymphoblastoid cell lines (405 siblings using Affymetrix HG U133 Plus 2.0 chips and 550 siblings using Illumina Human6V1 array)^40^ and 278 lung tissue samples from the Genotype-Tissue Expression (GTEx) project (http://www.gtexportal.org/home/), which provides a scientific resource to study human gene expression and regulation and its relationship to genetic variation^41^.

## Additional Information

**How to cite this article**: Wang, C. *et al*. The study of the relation of DNA repair pathway genes SNPs and the sensitivity to radiotherapy and chemotherapy of NSCLC. *Sci. Rep.* **6**, 26526; doi: 10.1038/srep26526 (2016).

## Supplementary Material

Supplementary Information

## Figures and Tables

**Table 1:** The details of SNPs related with DNA-repaired.

	SNP name	Gene name	Repair pathway	Reference
1	rs11615	ERCC1	NER	Dong , Hu Z, *et al*.^42^
2	rs2228000	XPC	NER,DR	Dong , Hu Z, *et al*.^42^
3	rs2228001	XPC	NER,DR	Vogel U, Overvad K, *et al*.^43^
4	rs1042522	TP53	NER,BER,DR	Lind H, Ekstrom PO, *et al*.^44^
5	rs2909430	TP53	NER,BER,DR	Mechanic LE, Bowman ED, *et al*.^45^
6	rs159153	OGG1	BER,NER	Liu X, Zhao J, *et al*.^46^
7	rs25489	XRCC1	BER	Burri RJ, Stock RG, *et al*.^47^
8	rs25487	XRCC1	BER	Giachino DF, Ghio P, *et al*.^48^
9	rs2273953	TP73	MMR	Liu L, Wu C, *et al*.^34^
10	rs2853677	TERT	DR	Van Dyke AL, Cote ML, *et al*.^49^
11	rs2736098	TERT	DR	Choi JE, Kang HG, *et al*.^50^
12	rs25406	PCNA	DR BER MMR	Rajaraman P, Hutchinson A, *et al*.^51^
13	rs8178085	DNA-PKcs	DR, NHEJ	Hu Z, Liu H, *et al*.^24^
14	rs12334811	DNA-PKcs	DR, NHEJ	Hu Z, Liu H, *et al*.^24^
15	rs1042858	RRM1	DR	Han JY, Yoon KA, *et al*.^52^
16	rs2279744	MDM2	DNA damage checkpoint\DNA damage response	Zhang X, Miao X, *et al*.^53^
17	rs10937405	TP63	DNA damage checkpoint\DNA damage response	Miki D, Kubo M, *et al*.^54^
18	rs1801270	CDKN1A	DNA damage checkpoint\DNA damage response	Själander A, Birgander R, *et al*.^55^
19	rs2735343	PTEN	DNA damage repair\DNA damage response	Jang Y, Lu SA, *et al*.^56^
20	rs2299939	PTEN	DNA damage repair\DNA damage response	Pu X, Hildebrandt MA, *et al*.^38^
21	rs8073069	survivin	DNA damage repair	Dai J, Jin G, *et al*.^57^
22	rs2132572	IGFBP-3	DNA damage repair	Han SG, Park KH, *et al*.^58^
23	rs344781	PLAUR	Related to DNA damage repair	Shih CM, Kuo WH, *et al*.^59^
24	rs1130214	akt1	Related to DNA damage repair	Pu X, Hildebrandt MA, *et al*.^38^
25	rs402710	CLPTM1L	Related to DNA damage repair	Yang P, Li Y, *et al*.^60^
26	rs2699887	PIK3	Related to DNA damage repair	Pu X, Hildebrandt MA, *et al*.^38^

DR, direct repair; NER, Nucleotide Excision Repair; BER, base excision repair; MMR, mismatch repair; NHEJ, Non-homologous end joining.

**Table 2:** SNPs related with sensitivity to radio-chemotherapy in squamous cell carcinoma.

Type	Gene	SNP	Genotype	case	β	SError	Chi square	Pr (>|z|)	OR
Chemotherapy	XPC	rs2228000	TT/TC	44	0				1
			CC	35	1.37218	0.533381	6.618267	0.010094	3.943933
	XPC	rs2228001	CA	35	0				1
			CC	10	1.81918	0.8732	4.340363	0.037219	6.166825
	TP73	rs2273953	CC	44	0				1
			TT	5	2.872139	1.283755	5.005493	0.025267	17.67478
	MDM2	rs2279744	GG/TG	64	0				1
			TT	15	1.512488	0.669008	5.111178	0.023772	4.538006
	PTEN	rs2299939	CC	48	0				1
			AA/CA	31	1.456732	0.567494	6.589272	0.01026	4.29191
	DNA-Pkcs	rs1234811	AA/AG	64	0				1
			AG	15	1.440435	0.624202	5.32521	0.021019	4.222532
Radiotherapy	DNA-PKcs	rs8178085	A	82	0				1
			CA	6	2.250114	1.047174	4.617121	0.031654	9.488816
	DNA-PKcs	rs12334811	AA/GG	69	0				1
			AG	19	1.603029	0.800815	4.006994	0.045312	4.96806

**Table 3:** SNPs related with sensitivity to radio-chemotherapy in adenocarcinoma.

Type	Gene	SNP	Genotype	case	β	SError	Chi square	Pr (>|z|)	OR
Chemotherapy	PIK3	rs2699887	AG	8	0				1
			GG	45	2.706417	1.151846	5.520785	0.018792	14.97553
	DNA-PKcs	rs12334811	AG	12	0				1
			AA/GG	41	1.532926	0.759122	4.077733	0.043452	4.631708
Radiotherapy	PTEN	rs2299939	AA/CA	23	0				1
			CC	34	2.749679	1.080702	6.473686	0.010948	15.63761
	PTEN	rs2735343	GG/CG	41	0				1
			CC	16	1.81269	0.835926	4.702331	0.030122	6.126925

**Table 4:** Correlation analysis between SNPs and the survival time of patients in squamous cell carcinoma and adenocarcinoma.

Type	SNP name	Gene name	Genotype	case	Progression-free survival (%)			cases	Overall survival (%)		
					1 year	3 years	P value		1 year	3 years	P value
Squamous cell carcinoma	rs2273953	TP73	CC	46	58.7	15.22		49	81.63	22.45	
			TT	7	42.86	0	0.072	6	66.67	0	0.027
			TC	33	51.52	0	0.037	36	80.56	19.44	0.168
	rs344781	PLAUR	CC	23	43.48	8.7		25	76	20	
			CT	37	56.76	8.11	0.265	38	78.95	15.79	0.65
			TT	24	62.5	8.33	0.037	27	85.19	22.22	0.125
	rs12334811	DNPK1	AG	15	40	6.67		19	73.68	10.53	
			AA	1	100	0	0.893	1	100	100	0.26
			GG	70	57.14	8.57	0.236	72	81.94	20.83	0.004
Adenocarcinoma	rs402710	CLPTM1L	CC	27	44.44	7.41		30	73.33	13.33	
			TT	4	25	0	0.02	5	80	0	0.06
			TC	27	33.33	11.11	0.022	29	72.41	24.14	0.479
	rs1801270	CDKN1A	AA	16	56.25	12.5		17	88.24	23.53	
			CC	19	26.32	10.53	0.037	23	60.87	13.04	0.143
			CA	21	33.33	4.76	0.148	22	72.73	13.64	0.581
	rs2279744	MDM2	GG	18	16.67	5.56		19	63.16	10.53	
			GT	28	46.43	10.71	0.03	29	82.76	24.14	0.033
			TT	12	50	8.33	0.023	16	68.75	12.5	0.731
	rs2909430	TP53	AA	53	39.62	9.43		59	74.58	16.95	
			AG	5	20	0	0.016	5	60	20	0.357

**Table 5:** 10 SNPs and gene expression in peripheral blood samples.

SNP	Chr	Allele	Effect Allele	*P* value	FDR	Gene	Cis-Trans
rs1130214	14	C/A	A	1.92E-43	0	AKT1	cis
rs1130214	14	C/A	A	5.99E-29	0	AKT1	cis
rs2228001	3	T/G	G	1.32E-28	0	XPC	cis
rs2132572	7	C/T	T	5.18E-18	0	IGFBP3	cis
rs159153	3	T/C	C	4.13E-12	0	TTLL3	cis
rs2228000	3	A/G	A	6.36E-12	0	XPC	cis
rs1130214	14	C/A	A	1.19E-11	0	SIVA1	cis
rs2228000	3	A/G	A	1.89E-10	0	TMEM43	cis
rs2228001	3	T/G	G	1.89E-08	0	TMEM43	cis
rs159153	3	T/C	C	1.91E-08	0	CAMK1,OGG1	cis
rs25489	19	C/T	T	3.99E-07	1.09E-04	PLAUR	cis
rs11615	19	A/G	G	6.74E-07	2.14E-04	ERCC1	cis
rs25489	19	C/T	T	1.07E-05	4.79E-03	PHLDB3	cis
rs2132572	7	C/T	T	1.59E-05	7.39E-03	IGFBP3	cis
rs159153	3	T/C	C	5.27E-05	2.23E-02	BRPF1	cis
rs1042522	17	C/G	G	7.36E-05	2.97E-02	CYB5D1	cis
rs344781	19	T/C	C	8.38E-05	3.32E-02	PLAUR	cis
rs344781	19	T/C	C	6.95E-04	1.89E-01	PLAUR	cis
rs1130214	14	C/A	A	8.03E-04	2.10E-01	SIVA1	cis
rs11615	19	A/G	G	9.11E-04	2.28E-01	KLC3	cis
rs159153	3	T/C	C	1.55E-03	3.22E-01	CRELD1	cis
rs2299939	10	C/A	A	2.10E-03	3.84E-01	PAPSS2	cis

**Table 6:** Three SNPs and gene expression in lymphoblastoid cell lines.

SNP	Ch	Allele1	Allele2	Frequency_Allele1	Effect_size	*P* value	Gene
rs2228001	3	G	T	0.424	−0.237	8.64E-32	XPC
rs25406	20	A	G	0.439	0.248	7.91E-25	C20orf30
rs8073069	17	G	C	0.685	0.21	6.89E-37	BIRC5
rs8073069	17	G	C	0.685	0.21	1.32E-32	BIRC5
